# Belfast Lunatic Asylum and the Chaplaincy Question

**Published:** 1853-04-01

**Authors:** 


### BELFAST LUNATIC ASYLUM AND THE CHAPLAINCY QUESTION.

In another portion of our columns to-day, says the Editor of the *Belfast Commercial Chronicle*, March 5th, will be found the official proceedings which have taken place between the Board of Governors of the above Institution and the Irish Executive, in connexion with the high-handed attempt on the part of the latter to force chaplains upon the former. Those proceedings are now presented authoritatively to the general public, a loud call being made for their production in consequence of the intense interest excited by the extraordinary, we might say most unconstitutional and arbitrary conduct of the Government, so transparent throughout this entire affair. We really are amazed beyond expression, now that we have before us the documents we this day publish, at the complete want of common sense, to say nothing of common prudence, displayed by those at the head of affairs in this country as relates to this matter. They would appear by their whole course, past and present, in regard to it, to be determined to fulfil to the letter the truthfulness of the expression so perfectly apt on the present occasion—*quem Deus vult perdere prius dementat.* Throughout the whole of this controversy we never had any other opinion, but that the governors of the asylum were in a right, and their autocratic opponents in a false, position. We formed this judgment from the ever able and admittedly satisfactory manner in which they discharged their high and important trust; for we challenge it to be said to the contrary, even by the most factious *leaguer*, that during the twenty-four years this institution has been in operation, a single complaint was ever made against any part of its management. So much to the reverse has this been the case that its system of general government has been the theme of universal approbation, and lauded to the utmost, and this not more so than deserved, by all parties, official and otherwise. Is it not then surprisingly strange, most monstrous, in fact, that those vested with a portion of the powers of her Majesty, to be exercised for the promotion of the common weal, should pervert those powers by wielding them in violation of the rules *enacted by themselves*, for the due and proper discipline of an invaluable public establishment like our district asylum? And yet this is palpably the case, as we shall now prove. On referring to the Board's remonstrance, it will be seen that a code of rules and regulations for the government of lunatic asylums in Ireland was deliberately made, sanctioned, and ordered to be carried into effect, by the Lord Lieutenant and Privy Council, in Ireland, in the year 1843—a code which, be it specially remembered, has, up to the present moment, been *unrepealed.* According to rule 13 of that code, then, we find it laid down that the "Board is to make rules and regulations for the admission of clergymen to visit in that character any patients of their own persuasion. Frequent visits of the parochial clergy to the institution are particularly desired; the Board to make arrangements for the celebration of divine service before such of the inmates as their respective clergymen and the physician shall deem fit to attend the same." This rule, so distinct and specific, is a finisher to this unparalleled attempt, and gross jobbing on the part of Government, to saddle chaplains upon this *or any other asylum* in Ireland. The Board state with respect to it, "We *have* made regulations for the admission of clergymen—we *have* encouraged the visits of the parochial and other clergy—we *have* made arrangements for the celebration of divine service before such of the inmates as their respective clergymen and the physician shall deem fit to attend the same; and being convinced that our arrangements were as judicious as they have unquestionably been salutary, we entreat your Excellency to sustain us in our disinterested and conscientious endeavours to carry them out as heretofore." In a foregoing part of this "remonstrance" our readers will see that in the year 1834 the above provisions were made by the Governors, the meeting at which the same were specially enacted being presided over by the late distinguished Bishop of the Diocese, and attended by the late most worthy and venerated Vicar, the Rev. A. C. Macartney, in addition to another estimable clergyman, the Rev. Thomas Hincks, still, happily, a zealous labourer in the vineyard, and long to continue so, we earnestly trust. Another of those still existing orders in Council—rule 45—is worthy, too, of the best attention. It is as follows—"The physician is to direct the course of moral and medical treatment of the patients;" the Board's remarks upon which being so pertinent that we cannot avoid reproducing them here—

"And assuredly no portion of moral treatment is so delicate and so important as that connected with religious teaching and ordinances.

"On behalf of our resident and visiting physicians, therefore, whose fidelity has been above all praise, and whose labours have been admittedly crowned with unequalled success, we implore your Excellency not to humiliate them by disregarding their matured opinions, or to paralyze their future exertions by permitting any novel interference with their professional arrangements.

"We respectfully assure your Excellency, that with the exception of the Lord Bishop of Down, the Right Rev. Dr. Denvir, the Rev. Dr. Edgar, and the Rev. Mr. Monsell, we have not conversed with any individual of any church, profession, or employment, who does not participate in our sentiments upon this question, and the ardour with which it has been opposed by newspapers representing, we believe, all creeds and parties, abundantly testifies the deep interest which it has awakened in the public mind.

"With regard to ourselves, we venture to assure your Excellency that, cheerfully admitting the sincerity of those who dissent from our views, we do not yield to them in our Christian anxiety to see the wounded spirit cheered and sanctified in due time and place by the blessed influence of religion, and we trust that our earnest pertinacity in this matter will be ascribed not to any morbid craving for victory or power, but to its true cause—an imperative sense of personal and public duty."

But there is one portion of this remonstrance which, above all others, must satisfy the most prejudiced, yea even our Tenant League contemporary, the *Banner*, that the conduct of our District Asylum has been so pre-eminently successful in the treatment of its inmates, that any interference with it would be the act of one "next door to an idiot." We now refer to that clause of it which gives a comparative statement of the cured and improved cases, taken, too, from the official report of Government, as made to Parliament, in reference to District Asylums, all of them fully chaplained, be it observed, except Belfast; according to which it will be seen that our own asylum is at the top of the scale, distancing all the others to such an extent, that they may be said to be *nowhere*. Here is a stubborn, here is a great fact, which our "worthy inspectors," though announced by the *Banner*—a bad authority, we admit—to be of such "professional eminence, as to counterbalance all other medical authority" (!!) never attempted to explain. Again, we find the inspectors duly recording, time after time, that the management, "*in detail*," of our asylum demanded the expression of their "unqualified satisfaction." Time and space, however, prevent us from entering more at length into this most able and convincing public document; every line of which is telling, and the whole scope of which is creditable in the highest degree to the governors. We must not, however, forget to direct the attention of all to the paragraph in it respecting the opinions of the physicians, past and present, of the asylum itself, on the subject of the systematic performance of religious services by chaplains. Such testimony, we submit, is most conclusive, if the judgment of men so highly qualified and experienced, whose talents and standing may, with every truth, be said—and this, too, without intending the slightest disrespect to our "worthy inspectors," (neither of whom, we believe, ever spent much time immediately with the insane, without which their wants could not be duly appreciated)—to be of "such professional eminence, as to counterbalance all other medical authority," however valuable.

Having now disposed of our cursory remarks upon the remonstrance of the board of governors, we cannot conclude without saying a word or two regarding the authorities by which it was accompanied, in support of the views it contained. And first, we beg to refer to the letter of the resident superintendent of the Armagh District Lunatic Asylum—a gentleman, whose opinions range over no less a period than thirty-four years in actual residence amongst the insane. Is the opinion of such a man not to be respected and acted upon? If so, there is no further need of arguing so important and vital a question. Then we have extracts from medical publications of the highest character, such as the *Dublin Quarterly Medical Journal*—the exponent of the opinions of the heads of the profession in Ireland, all expressing the strongest and most decided opinions against the so-called "medical aids," to wit, chaplains in hospitals for the insane. And though last, not least in importance, as regards our references to the papers now put forth by the governors, we beg very especially to direct general notice to the strikingly valuable and matured opinions of the Rev. William M'Ilwaine (for nearly eighteen years the incumbent of St. George's church, in this town), a christian minister, "whose praise is in all the churches," and whose practical piety and moral worth, as evidenced by his consistent daily walk in life, and the devoted attachment of his flock, none will presume to gainsay; opinions which, we hesitate not to affirm, are as correct as they are argumentative, and most creditable to him, both as a minister of the Gospel and a man. Another very remarkable evidence against chaplains is contained in the short but speaking extract from the letter of an actual chaplain—and in England too—whose testimony is founded on the practice of "experimental religion," to deny which to "dying lunatics!" some parties appear so monstrously shocked at.

But we have extended our remarks to a length which the really important nature of our subject has unwittingly led us on to, and, though much remains yet to be said, we must defer doing so to another opportunity. In the mean time we may state, as a very important fact in the present position of this chaplain question, that the grand jury of the county Down, at the assizes, now going on, have adopted, *unanimously*, the following resolution, strongly supporting the steps adopted by the authorities of the asylum against chaplains being permitted within its walls, a course which we take for granted the grand juries of the county Antrim, and the county of the town of Carrickfergus will likewise adopt:

*Grand Jury Room*, *Downpatrick*, 2*d March*, 1853.

A copy of a communication lately submitted to his Excellency the Lord Lieutenant from the governors of the Belfast District Lunatic Asylum, remonstrating against the appointment of chaplains, having been laid before the grand jury, together with the written opinions of eminently qualified medical practitioners, the matter was fully considered and discussed by the jury, and it was unanimously

"Resolved—That we highly approve and cordially concur in the views which have induced the governors to resist the appointment of chaplains, which, from the statements and opinions adduced, would, in our opinion, be seriously prejudicial to the recovery of the patients.

(Signed) "S. D. Crommelin, Foreman."

We have again to repeat, before concluding, that this chaplain agitation has stirred up a spirit and a feeling in the north of Ireland, which will not be quickly allayed. We presume that the Government never reckoned upon the old house they were bringing about their heads, in acting as they have done. We tell them plainly, they will find that this portion of Ulster is "fierce when provoked," and will not submit to be trampled upon, or ignominiously compelled to do their bidding, in order to make places for poor clerics, "with large families and small incomes," or to please grasping tenant-leaguers, or any other party—whether mitred, or cassocked, or gowned, or otherwise. No, we are not yet arrived at such a pitch of abject slavery as this, and with the blessing of Providence we never shall. The governors of the asylum, we must again remark, deserve all praise, for the independent conduct they have pursued—conduct not less respectful than determined; they have but to follow it up, and they may rest assured of success, and of the good opinion of all that is good in the community.

